# Effects of Environmental Conditions on Nephron Number: Modeling Maternal Disease and Epigenetic Regulation in Renal Development

**DOI:** 10.3390/ijms22084157

**Published:** 2021-04-16

**Authors:** Lars Fuhrmann, Saskia Lindner, Alexander-Thomas Hauser, Clemens Höse, Oliver Kretz, Clemens D. Cohen, Maja T. Lindenmeyer, Wolfgang Sippl, Manfred Jung, Tobias B. Huber, Nicola Wanner

**Affiliations:** 1 III Department of Medicine, University Medical Center Hamburg-Eppendorf, 20246 Hamburg, Germany; la.fuhrmann@uke.de (L.F.); o.kretz@uke.de (O.K.); m.lindenmeyer@uke.de (M.T.L.); t.huber@uke.de (T.B.H.); 2 Department of Medicine IV, Faculty of Medicine, University of Freiburg, 79106 Freiburg, Germany; saskia.lindner@googlemail.com (S.L.); clemens.hoese@uniklinik-freiburg.de (C.H.); 3 Institute of Pharmaceutical Sciences, University of Freiburg, 79104 Freiburg, Germany; alexander.hauser@pharmazie.uni-freiburg.de (A.-T.H.); manfred.jung@pharmazie.uni-freiburg.de (M.J.); 4 Nephrological Center, Medical Clinic and Policlinic IV, University of Munich, 80336 Munich, Germany; Clemens.Cohen@klinikum-muenchen.de; 5 Institute of Pharmacy, Martin-Luther-University of Halle-Wittenberg, 06120 Halle (Saale), Germany; wolfgang.sippl@pharmazie.uni-halle.de; 6 CIBSS—Centre for Integrative Biological Signalling Studies, University of Freiburg, 79104 Freiburg, Germany

**Keywords:** renal development, nephron number, diabetic nephropathy, epigenetic regulation, iron deficiency, DNA methylation

## Abstract

A growing body of evidence suggests that low nephron numbers at birth can increase the risk of chronic kidney disease or hypertension later in life. Environmental stressors, such as maternal malnutrition, medication and smoking, can influence renal size at birth. Using metanephric organ cultures to model single-variable environmental conditions, models of maternal disease were evaluated for patterns of developmental impairment. While hyperthermia had limited effects on renal development, fetal iron deficiency was associated with severe impairment of renal growth and nephrogenesis with an all-proximal phenotype. Culturing kidney explants under high glucose conditions led to cellular and transcriptomic changes resembling human diabetic nephropathy. Short-term high glucose culture conditions were sufficient for long-term alterations in DNA methylation-associated epigenetic memory. Finally, the role of epigenetic modifiers in renal development was tested using a small compound library. Among the selected epigenetic inhibitors, various compounds elicited an effect on renal growth, such as HDAC (entinostat, TH39), histone demethylase (deferasirox, deferoxamine) and histone methyltransferase (cyproheptadine) inhibitors. Thus, metanephric organ cultures provide a valuable system for studying metabolic conditions and a tool for screening for epigenetic modifiers in renal development.

## 1. Introduction

Fetal development is affected by the in utero environment, and an adverse milieu can predispose to diseases such as hypertension, cardiovascular disease and chronic kidney disease later in life [1,2,3,4]. A range of intrauterine disturbances can result in a reduction in nephron endowment and compromised renal function in the offspring. In rodents, conditions leading to reduced nephron numbers at birth include intrauterine growth restriction (IUGR), maternal low protein diet, medications (including corticosteroids or nonsteroidal anti-inflammatory drugs), monogenetic mutations and low vitamin A levels, as well as maternal diabetes and iron deficiency [5,6,7,8,9,10]. In humans, there is currently no noninvasive method of measuring nephron numbers. However, postmortal studies have demonstrated a negative correlation between nephron numbers and blood pressure [4,11]. Additionally, intrauterine conditions associated with reduced nephron numbers such as IUGR are known to be are associated with increased rates of hypertension and CKD [12].

In vivo experiments where the adverse intrauterine conditions are artificially induced in pregnant animals have proven invaluable for the detection and description of the renal alterations induced in the offspring. However, any experimental intervention during gestation results in complex alterations in the maternal, placental and fetal physiology which may themselves affect the environment of the developing metanephroi. Ex vivo modeling techniques circumvent this by enabling the investigation of kidney development completely separated from the influence of the mother animal, the placenta or other organs of the fetus. Isolated cultures of metanephroi on a medium–air interface were initially performed by Trowell in 1950 [13] and have subsequently been refined by culturing of kidneys on filter membranes [14], providing a basis for single-variable culture conditions.

In summary, there is an increasing body of evidence suggesting that prenatal insults associated with low nephron numbers are relevant risk factors for hypertension and CKD. In order to develop preventative strategies to ensure adequate nephron endowment at birth, a mechanistic understanding of the factors influencing renal development and nephrogenesis is necessary. In the present work, metanephric organ culture was used to model different aspects of environmental regulation to study their effects on renal growth and possible implications for long-term renal function and used to screen for epigenetic regulators using FDA-approved small compounds.

## 2. Results

### 2.1. Use of Metanephric Organ Cultures to Study the Effect of Environmental Conditions on Renal Development

**Figure 1:**
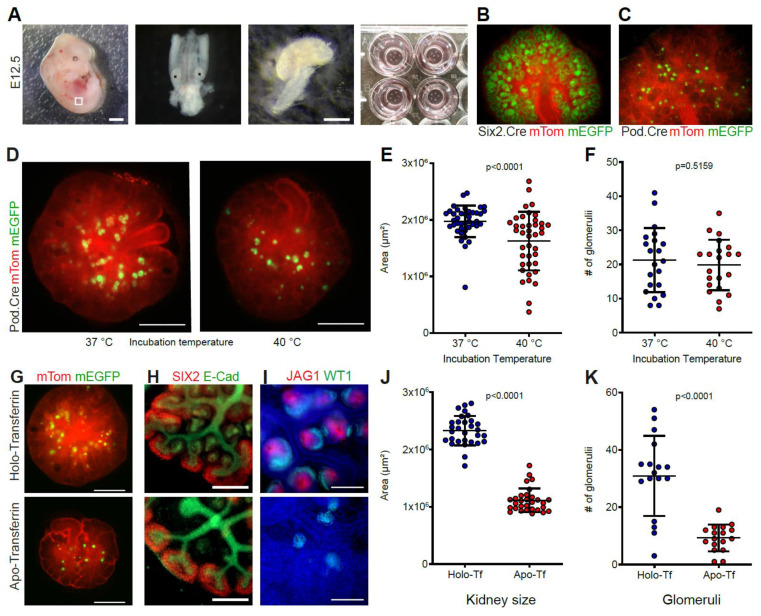
Use of metanephric organ cultures to study the effect of environmental conditions on renal development. (**A**) The urogenital ridge from E12.5 mouse embryos (left panel) was microsurgically extracted (second panel), and the kidneys were isolated (third panel) and placed on Transwell inserts (right panel). Scale bars: 1 mm (left panel), 500 µm (third panel). (**B**) Transgenic mice with dual Tomato/EGFP expression were used for conditional labeling of Six2-positive cells and their offspring using Six2.Cre or (**C**) podocin-positive cells using Pod.Cre mice. (**D**) Explants from the same embryo cultured for 7 days at 37 or 40 °C. Scale bars: 500 µm. (**E**) Surface areas of explants grown for 7 days at 37 or 40 °C. n = 40 pairs, paired *t*-test, mean ± SD. (**F**) Number of glomeruli in the explant groups after 7 days. n = 21 pairs, paired *t*-test, mean ± SD. (**G**) Explants from the same embryo cultured for 7 days in medium containing holo-Tf or apo-Tf. Scale bars: 500 µm. (**H**) Widefield images of holo-Tf and apo-Tf cultured explant pair stained against SIX2 and E-cadherin after 48 h of culture show normal progenitor cell pool and defects in early nephron morphology. Scale bar: 100 µm. (**I**) Widefield images of holo-Tf and apo-Tf cultured explant pair stained against WT1 and JAG1. Scale bars: 100 µm. (**J**) Surface areas of holo-Tf and apo-Tf cultured explants after 7 days. n = 30 pairs, paired *t*-test, mean ± SD. (**K**) Number of glomeruli in the explant groups after 7 days. n = 17 pairs, paired *t*-test, mean ± SD.

To model adverse environmental conditions, kidneys from embryonic day (E) 12.5 embryos were cultured at the medium–air interface (Figure 1A). To facilitate monitoring of nephron and glomerular development, Six2.Cre and Pod.Cre dual-fluorescent reporter mice were used, respectively (Figure 1B,C). A common condition during pregnancy is fever, which affects more than 10% of pregnancies during the first 16 weeks of gestation [15]. Heat is a well-characterized teratogen, and hyperthermia during pregnancy has been shown to lead to fetal abortion, growth retardation and developmental defects, such as renal agenesis, hypoplasia and low birth weight in several species [16,17,18,19,20,21]. To assess the impact of prolonged, fever-range hyperthermic conditions on kidney growth and nephron formation, kidneys were isolated and maintained at either 37 or 40 °C (Figure 1D). After 7 days of culture, kidneys cultured at 40 °C were, on average, 18.36% smaller than their counterparts cultured under physiological conditions (Figure 1E). However, no significant difference in the number of glomeruli per kidney was found between the groups (Figure 1F). Nevertheless, the decreased overall growth of the kidneys demonstrates a negative effect of increased temperature on metanephric growth.

With an estimated 19% of pregnant women suffering from iron-deficiency anemia [22], iron deficiency is one of the most widespread conditions with the potential to disturb renal development [23]. In vivo data have shown that renal growth, glomerular numbers and renal iron uptake are reduced during pregnancies affected by maternal iron deficiency [24]. In order to assess the impact of reduced transferrin-bound iron supply on kidney growth and nephrogenesis, explants were cultured in medium containing 50 µg/mL of iron-saturated holo-transferrin or 50 µg/mL of iron-depleted apo-transferrin, respectively. Iron-restricted kidneys remained much smaller than their iron-sufficient counterparts and showed increased apoptosis in the ureteric buds and reduced ureteric bud branching and proliferation (Figure 1G, Supplementary Figure S1A–F). While the nephron population was morphologically unaffected, a decrease in the developing distal part of the nephron, as well as distal tubules, could be seen (Figure 1H,I, Supplementary Figure S1G–J). The iron-deficient kidneys were, on average, 47.9% of the size of their holo-transferrin cultured counterparts (Figure 1J) and showed a reduction in the overall number of glomeruli per kidney of 69.9% after 7 days in culture (Figure 1K). Thus, iron depletion by apo-transferrin showed severe effects on kidney growth with an all-proximal nephrogenesis phenotype.

### 2.2. Ex Vivo High Glucose Exposure Leads to Diabetic Nephropathy-Associated Changes in the Developing Kidney

**Figure 2:**
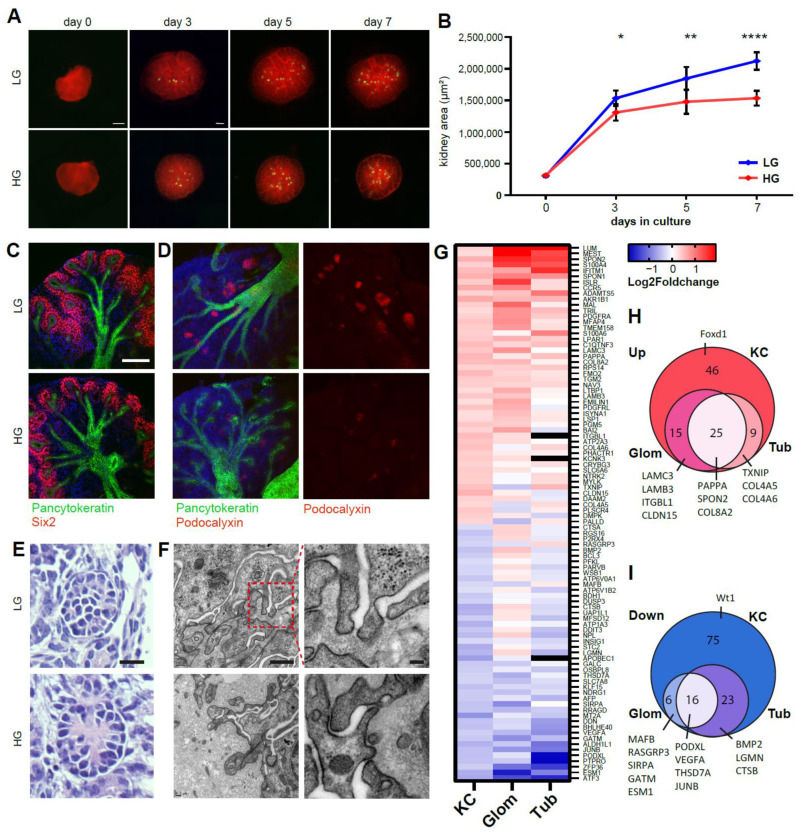
Ex vivo high glucose exposure leads to diabetic nephropathy-associated changes in the developing kidney. (**A**) Embryonic kidneys from Pod.Cre;Tomato/EGFP animals cultured for 7 days in low glucose (LG, 5.5 mM α-D-glucose, 55 mM mannitol) or high glucose (HG, 55 mM α-D-glucose) conditions. Scale bar: 500 µm. (**B**) Kidney surface area of HG and LG conditions. *, *p* = 0.0474; **, *p* = 0.0052; ****, *p* < 0.0001. Paired *t*-test, mean ± SD. (**C**) Confocal immunofluorescent stainings of day 7 kidney cultures against Six2 and (**D**) podocalyxin with pan-cytokeratin and Hoechst. Scale bar: 100 µm. (**E**) Stainings of 6 µm sections from day 7 kidney cultures. Scale bar: 20 µm. (**F**) Transmission electron microscopy of sections from day 7 kidney cultures. Glomerular basement membranes are thickened in kidneys exposed to high glucose conditions. Left column: magnification showing podocyte foot processes. Scale bars: 500 nm (left panels), 100 nm (right panels). (**G**) Fold change of RNA-seq data from HG compared to LG kidneys and ERCB diabetic nephropathy (DN) patient microarray data from microdissected glomeruli and tubules showing differentially expressed genes. (**H**) Genes upregulated in the kidney cultures (KC) overlapping with ERCB DN patient data and selected genes highlighted. (**I**) Genes downregulated in the kidney cultures (KC) overlapping with ERCB DN patient data and selected genes highlighted.

Maternal diabetes is another common condition during pregnancy, with a global prevalence of hyperglycemia in pregnancy of ~17% and over 20 million live births each year [25]. Diabetes induced in mouse and rat models has been shown to lead to offspring with a lower nephron number [10,26,27]. Metanephric organ culture has been used before to study the effect of high glucose on renal development [27,28,29]. Previously, we reported reduction in size, nephron number and DNA methylation under high glucose conditions of 55 mM [30]. In contrast to published data [28], no effect on explant size or glomerular number could be seen in our samples when cultured in different 30 mM glucose media compared to 5 mM control conditions after 7 days (Supplementary Figure S2A,B). Furthermore, no decrease in DNA methylation at LINE-1 and major satellite sites could be detected (Supplementary Figure S2C,D). The effect of 55 mM high glucose on renal development after a 7-day period culture was further analyzed, showing a decrease in the growth rate starting at day 3 in culture (Figure 2A,B). Immunofluorescence stainings showed no morphological defects of the SIX2-positive progenitor cell pool (Figure 2C) but showed reduced staining of podocyte marker podocalyxin (PODXL, Figure 2D). The glomeruli were found to contain a thickened glomerular basement matrix visible in histological stainings (Figure 2E). Similar findings were made in electron microscopy, showing an increase in glomerular basement membrane thickness (Figure 2F), one of the earliest markers of prediabetes and diabetic nephropathy (DN) [31,32], in five out of six kidneys and none of seven littermate control kidneys. To further unravel changes in the transcriptome, pairwise differential gene expression (DGE) analysis of kidney cultures from three litters was performed, with one kidney from each embryo cultured with high glucose and the other with control medium and the kidneys pooled for analysis (n = 3). DGE confirmed the upregulation of extracellular matrix components as the primary upregulated biological process (Supplementary Table S1). Downregulated genes were mainly involved in (immune) cell activation and exocytosis/secretion (Supplementary Table S1). Mammalian phenotype ontology indicated abnormal kidney cortex and renal corpuscle morphology due to downregulated genes such as Pdgfb, Podxl, Ren, Ptpro, Mafb and Vegfa (Supplementary Table S1). Renal expression of several genes, such as Angptl4, Spon2 (Mindin), Pappa and Txnip, which have been shown to be upregulated in diabetic nephropathy [33,34,35,36], was found to be increased under hyperglycemic conditions. To compare the high glucose kidney culture gene expression profile to human DN, the DGE data were matched to human data from microdissected glomeruli and tubules from diabetic nephropathy patient biopsies from the European Renal cDNA Bank (ERCB). From the 216 differentially regulated genes matched after batch analysis, 94 genes were correspondingly differentially regulated in the glomerular and/or tubular fractions (Figure 2G). The overlap of our model and human DN genes showed 40 out of 95 genes upregulated in the glomeruli and 34 genes in tubules (25 genes in common) (Figure 2H). The genes were mostly involved in extracellular matrix organization (COL4A5, COL4A6, COL8A2, LAMB3, LAMC3) and cell adhesion (ITGBL1, CLDN15). Additionally, diabetes-associated genes such as TXNIP, SPON2 and PAPPA were upregulated. Out of 120 downregulated genes from the kidney cultures, 22 were also downregulated in the glomeruli and 39 were also downregulated in the tubules (16 in common (Figure 2I). These genes were involved in response to endogenous stimulus (BMP2, KLF15, JUNB, CTSB), nephron epithelium development (PTPRO, PODXL, VEGFA) and positive regulation of endothelial cell chemotaxis (LGMN, P2RX4, VEGFA). Additionally, diabetes-associated genes such as RASGRP3, SIRPA, GATM and ESM1 were downregulated [37,38,39]. Differentially regulated genes not overlapping with ERCB data also reflected diabetes-associated changes, such as extracellular matrix (ANGPTL4, TNN, DPT, COL9A2) or gestational diabetes (LAT2, HP, CXCL10, CD86, CD68, REN, SLC2A3, VCAM1). Thus, renal development under high glucose conditions displayed remarkable similarities to human adult diabetic nephropathy.

### 2.3. Ex Vivo High Glucose Exposure Influences to Long-Term Memory Formation via DNA Methylation

**Figure 3:**
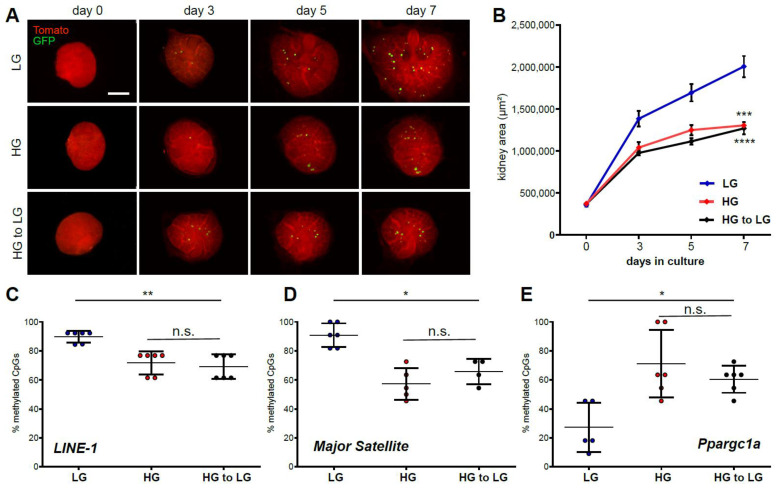
Ex vivo high glucose exposure influences long-term memory formation via DNA methylation. (**A**) Imaging of E12.5 embryonic kidneys from day 0 to day 7 in low glucose medium (5.5 mM), high glucose medium (55 mM) or high glucose medium for 3.5 days and reversal to low glucose medium for the remaining days. Scale bar: 500 µm. (**B**) Kidney surface area over 7 days. Mean ± SD. n = 36 kidneys. LG, low glucose treatment; HG, high glucose treatment; HG to LG, 3.5 days high and 3.5 days low glucose treatment. ***, LG–HG (unpaired *t*-test): *p* = 0.0004; ****, LG–HG to LG (paired *t*-test): *p* < 0.0001. (**C**) Analysis of the DNA methylation at *LINE*-1 and (**D**) *major satellite* loci shows continuous DNA hypomethylation in high glucose treated conditions. **, *p*-value = 0.0022. *, *p*-value = 0.0357. (**E**) Analysis of the DNA methylation at *Ppargc1a* locus shows continuous DNA hypermethylation in high glucose conditions. Mean ± SD. *, *p*-value = 0.0130.

To further understand the molecular changes mediated by a hyperglycemic environment, kidney cultures were grown at high glucose conditions for 3.5 days and then changed to low glucose conditions for the same amount of time (Figure 3A). Remarkably, incubation under physiological conditions after the shorter incubation period in high glucose medium did not reverse growth reduction after 7 days in culture with the cultures growing at the same rate as under continuous high glucose treatment (Figure 3B). Furthermore, DNA methylation showed hypomethylation of LINE-1 element and major satellite loci (Figure 2C,D), as well as sustained DNA hypermethylation of the Ppargc1a promoter, under both high glucose and reversed conditions (Figure 3E), indicating the formation of metabolic memory via DNA methylation due to the earlier adverse environmental conditions as a means of fetal programming.

### 2.4. Ex Vivo Small Compound Screen Identifies Epigenetic Regulators of Renal Development

**Figure 4:**
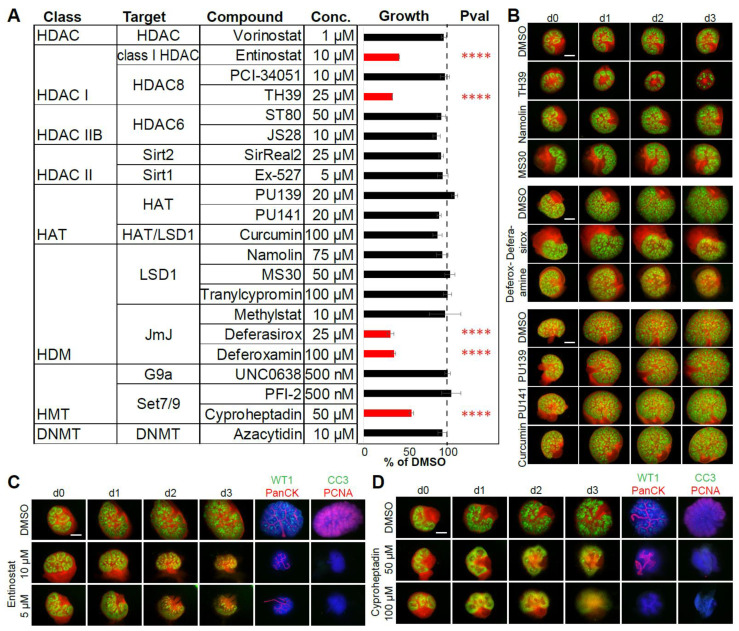
Ex vivo small compound screen identifies modulators of renal development. (**A**) List of small compounds, their epigenetic targets and concentration used shows renal growth reduction with entinostat, TH39, deferasirox, deferoxamine and cyproheptadine in ≥3 independent experiments after 3 days in culture. Control cultures were treated with DMSO. ****, *p*-value < 0.0001. (**B**) Examples of two sets of embryonic kidney cultures with pictures taken from day 0 until day 3 showing growth reduction and morphological differences in kidneys treated with TH39, deferasirox and deferoxamine compared to littermate control kidneys. Scale bar: 500 µm. (**C**) Entinostat showed growth reduction at 5 and 10 µM concentration, no nephron differentiation and lack of proliferation after 3 days. (**D**) Cyproheptadine showed growth reduction at 100 and 50 µM concentrations, lack of differentiation, ureter dilation and lack of proliferation after 3 days in culture. Scale bar: 500 µm. PanCK, pan-cytokeratin. CC3, cleaved caspase-3. PCNA, proliferating cell nuclear antigen.

The results of this work as well as previous works suggest that epigenetic mechanisms play a role in kidney development [30,40,41,42,43,44,45]. Therefore, we wanted to systematically evaluate the effect of epigenetic modulators of the different enzyme classes on renal development. For this, we selected a library of 22 FDA-approved small compounds with demonstrated inhibitory activity [46,47,48,49,50,51,52,53,54,55,56] (Figure 4A). Using Six2.Cre-reporter mice to evaluate nephron development, the renal structures were cultured for 3 days with the inhibitors in the medium. Size increase over time was compared to the littermate control organs, and the morphology was checked for abnormalities in development (Figure 4B). Several inhibitors could be shown to interfere with normal ex vivo renal development. HDAC inhibitor entinostat, a benzamide histone deacetylase inhibitor with high affinity for HDAC 1, 2 and 3 [57], showed consistent growth reduction and lack of differentiation and proliferation after 3 days (Figure 4C). TH39, developed as a selective HDAC8 inhibitor (IC_50_ HDAC8 88 nM, 26-fold selective against HDAC1, 28-fold selective against HDAC6 [56]), showed a similarly severe inhibition of growth compared to littermate control organs. Furthermore, iron chelators and inhibitors of JmJC deferasirox and deferoxamine showed growth reduction analogous to iron-deficient medium. Additionally, SET7/9 inhibitor cyproheptadine [58,59] showed growth reduction and lack of differentiation and proliferation compared to control kidneys (Figure 4D) but also seemed to interfere with Wnt signaling (Supplementary Figure S3). Other HDAC, HAT, HDM and HMT inhibitors and DNMT inhibitor 5-azacytidine did not show growth reduction or developmental anomalies within the measured time frame (Figure 4A).

## 3. Discussion

Renal development primarily takes place in utero and is subject to interference from metabolic and environmental influences. Nephron number is determined at birth, and a growing body of evidence suggests low nephron numbers to be a risk factor for the development of hypertension and chronic kidney disease later in life [2,3,4,60]. However, many factors influencing nephron number and modes of action are still unknown. Here, embryonic kidneys from dual-fluorescent reporter mice cultured on Transwell inserts were used to model maternal metabolic conditions and screen epigenetic inhibitors.

While many studies now involve kidney organoids using human iPS cells [61], kidney cultures are a valuable tool for detailed analysis of phenotypes mediated by metabolic conditions or inhibitory agents. While progenitor cell cultures and kidney organoids are alternatives with significant potential for investigations of teratogenicity, they are subject to limitations, such as high variability in differentiation and growth and lack of a conventional organ structure. This makes metanephric organ culture a valuable alternative to investigate conditions that would not be feasible in vivo.

Thus, hyperthermia and iron deficiency provide two examples of investigating environmental conditions with different outcomes on renal growth and nephron numbers. While data on the effect of hyperthermia on renal growth are scarce, the effect of ex vivo iron restriction is in line with previous reports of maternal iron deficiency in rats [23,24], although the model is limited in replicating the exact in utero state in terms of concentration and iron kinetics. Our results have also shown that mitotic activity is reduced in the iron-restricted condition. Iron plays an essential role in many cellular processes, such as the cell cycle [62]. Our morphological analysis showed an all-proximal nephron differentiation phenotype, possibly as the result of inhibited canonical Wnt signaling. Widespread downregulation of genes associated with Wnt signaling has previously been reported in a microarray analysis of rat offspring exposed to maternal iron deficiency [63]. Mechanistically, intracellular iron depletion by chelating agents has been shown to induce proteasomal degradation of β-catenin, the principal downstream effector protein of the canonical Wnt pathway in cancer and neural progenitor cells [64,65].

As another metabolic condition, modeling high glucose exposure resulted in effects similar to those of in vivo streptozotocin-induced maternal diabetes [10,26,27,66,67,68,69,70,71]. While our model could not reproduce the effects of 30 mM glucose conditions, likely due to the previously reported influence of mouse background [28,72], 55 mM glucose conditions resulted in growth reduction and decreased nephron numbers. Pronounced changes were visible histologically and ultrastructurally in the glomeruli, with the expansion of the glomerular basement matrix resembling human diabetic nephropathy. The similarities between murine fetal and human adult renal response to high glucose with distinct ECM expansion and downregulation of key podocyte genes was striking and revealed many known diabetes-associated genes, indicating usage of similar mechanisms in the podocytes and tubules. However, whether the same underlying pathways lead to dedifferentiation in diabetic nephropathy and decrease in differentiation during fetal differentiation is so far unknown. Interestingly, nonglucose alterations of the diabetic environment, such as hyperketonemia, have also been shown to mediate teratogenesis but were not replicated in the kidney culture model [73,74]. Long-term effects of hyperglycemia were also reproducible in our model, showing continuous growth retardation after normalization of glycemia similar to previous reports [10]. Additionally, DNA methylation analysis revealed a prolonged DNA hypomethylation at repetitive regions and hypermethylation at the *Ppargc1a* locus after reversal to low glucose medium, indicating the formation of metabolic memory after a period of metabolic stress [30]. Many more of the differentially regulated genes have also previously been shown to be epigenetically regulated in diabetes. For instance, upregulated gene *MEST* is maternally imprinted and hypomethylated in gestational diabetes mellitus [75]. *S100A4* is a differentially methylated marker of insulin resistance in obese children [76]. Of the downregulated genes, *ESM1* and *RASGRP3* are examples of genes differentially methylated in gestational diabetes [77,78], showing the growing emergence of links between diabetes and epigenetic regulation. Thus, our system highlights epigenetic modification or fetal programming as an important regulatory mechanism.

Several epigenetic regulatory enzymes are known to be involved in both renal morphogenesis and transcriptional regulation in adult organ function and disease [30,40,79,80,81]. Using kidney cultures as a screening tool, we searched for additional epigenetic modifiers playing a role in renal development and nephron morphogenesis. Entinostat (MS-275) reduced kidney size concordant with previously reported genetic deletion of HDAC1 and HDAC2 in either nephron progenitor cells or ureteric bud cells [44,82,83]. While inhibition of HDAC8 by TH39 also induced a severe growth reduction of the explant and lack of differentiation, the highly specific HDAC8 inhibitor PCI-34051 showed no effect, thus pointing to off-target effects of TH39, such as other HDACs. With a phenotype similar to that of kidneys cultured under iron-deficient conditions, deferasirox and deferoxamine, published inhibitors of the iron-dependent JumonjiC-domain-containing histone demethylases [50], may exhibit off-target effects due to iron chelation. While cyproheptadine, a histamine antagonist and published inhibitor of Set7/9 histone methyltransferase, displayed a unique phenotype by leading to diffusion of the progenitor cell population, additional results pointed to off-target effects involving the Wnt signaling pathway [48]. Altogether, this library of FDA-approved inhibitors shows the potential for fast and effective screening for epigenetic modulators. Due to the limited time frame used, inhibitors requiring prolonged exposure to exert an effect might have been missed in this setup. Thus, azacytidine did not show an effect despite the published phenotype of Dnmt1 knockout [30].

While the kidney culture system offers robust renal development, several aspects limit investigation or interpretation of the results in this reductionist model. First, studies have shown differences in mouse and human development and gene expression, limiting the use of the mouse model. Next, time limitations in this system may not allow for all complex processes to unfold. This may account for some of the environmental conditions and epigenetic inhibitors failing to impact renal development in this study. Moreover, some of the inhibitors show nonspecific effects that can contribute to inhibition of other pathways, such as in the case of cyproheptadine, which appears to exhibit its effect via activation of GSK3-beta, or the case of iron chelation by deferasirox/deferoxamine. Beyond this proof-of-concept study, the epigenetic inhibitor screen could be extended to more (specific) inhibitors; prolonged time periods; additional study criteria, such as nephron number and ureteric bud branching; and transcriptomic and epigenetic studies.

To summarize, kidney cultures enable the characterization of a number of maternal disease models, such as hyperthermia, iron deficiency and maternal diabetes, and the screening of pharmacological compounds, providing a well-suited platform for investigating the crosstalk between environmental influences of the developing kidney and its epigenetic programming.

## 4. Materials and Methods

### 4.1. Animal Handling

Mice were kept in a specific-pathogen-free environment at the Center for Experimental Models and Transgenic Service (CEMT) in Freiburg, Germany. All mice were raised in a 12/12 h cycle of light and darkness, with access to water and standard chow ad libitum. All experiments were registered with the regional government of Baden-Wuerttemberg under the authorization codes X15/03R and X17/05F.

### 4.2. Timed Harvest of Embryos/Microdissection/Culture of Metanephroi

Timed-pregnant hNPHS2Cre B6.129(Cg)-Gt(ROSA)26Sortm4(ACTB-tdTomato,-EGFP)Luo/J [5] and Tg(Six2-EGFP/cre)1Amc/J mice [84] were sacrificed at E12.5, and the embryos were harvested. The metanephroi were isolated and cultured on Transwell inserts with culture medium at the medium–air interface. Pairing of metanephroi from the same embryo between control and experimental conditions was maintained throughout the experiments unless mentioned otherwise.

### 4.3. Genotyping

Genotyping of the mice was performed by polymerase chain reaction (PCR) amplification of DNA isolated from tail biopsies and subsequent visualization of the amplified fragments by gel electrophoresis. The following primers were used: Tomato/EGFP forward 5′ CTC TGC TGC CTC CTG GCT TCT 3′ reverse wildtype 5′ CGA GGC GGA TCA CAA GCA ATA 3′ and reverse mutant 5′ TCA ATG GGC CGG GGT CGT T3′, Cre forward 5′ GCA TTA CCG GTC GAT GCA ACG AGT GAT GAG 3′ and reverse 5′ GAG TGA ACG AAC CTG GTC GAA ATC AGT GCG 3′.

### 4.4. Hyperglycemic Conditions

Hyperglycemia medium contained DMEM medium with 10% fetal bovine serum, 100 µg/mL of penicillin, 100 µg of streptomycin and D-glucose for a final concentration of 55 mM D-glucose. For the control medium, an equimolar amount of mannitol was added, for a final concentration of 5 mM of D-glucose and 50 mM mannitol.

### 4.5. Hyperthermic Conditions

For both hyperthermia and control cultures, a serum-free base medium of 1:1 DMEM and Ham’s F-12 Medium was supplemented with 100 µg/mL of penicillin, 100 µg/mL of streptomycin, 50 µg/mL of bovine holo-transferrin and 10 mM HEPES for increased buffering capacity. Control cultures were incubated at 37 °C, while hyperthermia cultures were incubated in an identical incubator at a temperature of 40 °C.

### 4.6. Iron-Restriction

Iron-restricted medium consisted of serum-free base medium of 1:1 DMEM and Ham’s F-12 medium which was supplemented with 100 µg/mL of penicillin, 100 µg/mL of streptomycin and 50 µg/mL of apo-transferrin. For the iron-sufficient control cultures, 50 µg/mL of holo-transferrin was used instead.

### 4.7. Whole Mount Immunofluorescence Staining of Explants

Cultured explants were either fixed with cold methanol for 20 min or room-temperature 4% paraformaldehyde (PFA) solution for 15 min and subsequently washed three times with room temperature PBST buffer (PBS + 0.1% Tween 20) for 5 min. Blocking solution containing 5% BSA in PBST buffer was added for 3 h at room temperature. After blocking, the cultures were incubated in dilutions of the primary antibodies in blocking solution at 4 °C on an orbital shaker overnight. Cultures were then washed three times with blocking solution for 2 h each and incubated in a 1:300 dilution of secondary antibodies and 1:500 dilution of Hoechst nuclear dye in blocking solution overnight. The cultures were again washed three times for 2 h and mounted with Prolong Gold Antifade mountant using a spacer. The following primary antibodies were used: rabbit anti-active caspase 3 (1:250, AF835; R&D systems Inc., Minneapolis, MN, USA), rabbit anti-JAG-1 (1:100, 260S; Cell Signaling Technology, Denver, MA, USA), sheep anti-Tamm-Horsefall protein (1:250, AB2606308; Thermo Fisher Scientific, Waltham, MA, USA), mouse anti-pan-cytokeratin (1:250, AB11213; Abcam, Cambridge, UK), rabbit anti-SIX2 (1:100, 11562-1-AP; Proteintech Group Inc., Manchester, UK), mouse anti-WT1 (1:100, 05-753; Merck KGaA, Darmstadt, Germany), rat anti-CD326 (1:100; 118202, Biolegend, San Diego, CA), rabbit anti-NKCC2 (1:100, SPC-401D; StressMarq Biosciences, Victoria, BC, Canada), mouse anti-phospho-histone H3 (1:100, 9706S, Cell Signaling Technology, Denver, MA, USA) and mouse anti-E-cadherin (1:200, 4A2C7; Thermo Fisher Scientific, Waltham, MA, USA). The following secondary antibodies were used in 1:300 dilution: Alexa Fluor 488 anti-mouse (R37114) and anti-rabbit (R37118); Alexa Fluor 555 anti-mouse (A-31570), anti-rabbit (A-31572), anti-sheep (A-21436) and anti-rat (A-21434) (all Thermo Fisher). The pairing of explants from the same embryo was not maintained in the stainings against phospho-histone H3 due to the explants detaching from the membrane during PFA fixation.

### 4.8. Imaging

Live imaging of cultured metanephroi from NPHS2-Cre;Tomato/EGFP mice was performed with Zeiss AxioObserver after mounting the membrane inserts on a glass-bottom dish containing 200 µL of cold PBS. Live images were taken as z-stacks with a plane distance of 10 µm. For the glomerular counting, the z-stacked GFP channels were orthogonally projected using Zen Blue and analyzed with ImageJ. Stained cultures were imaged using Zeiss AxioObserver inverted microscope or U2 LSM 510 META laser scanning microscope. Mitotic cells were counted using ImageJ.

### 4.9. Histology

Fixation was performed in 4% PFA solution overnight. The explants were dehydrated with ethanol, incubated with xylene and embedded in paraffin. The paraffin blocks were sectioned at 3 µm and mounted onto glass specimen slides. Hematoxylin/eosin staining (H&E) was performed.

### 4.10. EM

Kidney cultures were fixed in 4% PFA/1% glutardialdehyde in 1x PBS overnight and then embedded in liquid 40 °C agarose. After postfixation with 1% osmium tetroxide in 6.68% sucrose buffer, the samples were washed and stained en bloc with 1% uranyl acetate in 70% alcohol for 1 h, dehydrated in ethanol and propylene oxide and embedded in Durcupan (Plano, Wetzlar, Germany). Ultrathin sections were stained with lead citrate and examined in a Zeiss-Leo 910 transmission electron microscope.

### 4.11. Bisulfite-PCR

DNA from kidney cultures was isolated with DNeasy Blood & Tissue kit (QIAGEN) and bisulfite-converted using EpiTect Bisulfite Kit (QIAGEN) according to the manufacturer’s instructions. Converted DNA (20 ng) was used as template in a PCR reaction using AmpliTaq Gold Polymerase (Invitrogen). PCR products were purified using gel electrophoresis and ligated into a pCR4-TOPO vector using the TOPO TA Cloning Kit for sequencing (Invitrogen) and transformed into DH10B *E. Coli* cells. Randomly selected clones were sent for sequencing (GATC, Konstanz). Inspection, alignment, visualization and statistics were performed with QUMA: quantification tool for methylation analysis [85]. The following primers were used: major satellite forward: 5′ GGA ATA TGG TAA GAA AAT TGA AAA TTA TGG 3′, reverse: 5′ CCA TAT TCC AAA TCC TTC AAT ATA CAT TTC 3′, [30] Line-1 forward 5′ TAG GAA ATT AGT TTG AAT AGG TGA GAG GT 3′, Line-1 reverse: TCA AAC ACT ATA TTA CTT TAA CAA TTC CCA 3′, [30] Ppargc1a forward 5′ TGT TAG GGA ATA AGA TTT GTG TTT TTA A 3′, Ppargc1a reverse 5′ CAA ATA CTC CTA TAA ACA ATC CAA ACA A 3′.

### 4.12. RNA Sequencing

The RNA of kidneys grown for 7 days under high or low glucose conditions was isolated using the Qiagen RNeasy Plus Mini Kit. RNA sequencing was performed by GATC Biotech. Quality control was done with FastQC (Barbraham Bioinformatics). Raw reads were trimmed using TrimGalore! (Barbraham Bioinformatics) and mapped with Tophat (v2) [86] to mm10 using Galaxy Freiburg. Read counts were extracted with htseq-count [87]; differential gene expression was analyzed with DESeq2 [88].

### 4.13. Microarray Analysis of Human Kidney Biopsies

Human kidney biopsy specimens and Affymetrix microarray expression data were procured within the framework of the European Renal cDNA Bank–Kröner–Fresenius Biopsy Bank. Biopsies were obtained from patients after informed consent and with the approval of the local ethics committees [89]. Following a renal biopsy, the tissue was transferred to RNase inhibitor and microdissected into glomeruli and tubulointerstitium. Total RNA was isolated from microdissected glomeruli and tubules, reverse transcribed and linearly amplified according to a protocol previously reported [90]. In this study, we used published microarray expression data from individual patients with diabetic nephropathy, as well as living donors (GSE 99340, LDs: GSE32591, GSE35489, GSE37463). CEL file normalization was performed with the Robust Multichip Average method using RMAExpress (Version 1.0.5) and the human Entrez-Gene custom CDF annotation from Brain Array Version 18, 23 January 2014 (http://brainarray.mbni.med.umich.edu/Brainarray/Database/CustomCDF/CDF_download.asp). The log-transformed dataset was corrected for batch effect using ComBat from the GenePattern pipeline (version 3.8.0) (http://www.broadinstitute.org/cancer/software/genepattern/). To identify differentially expressed genes, the SAM (Significance Analysis of Microarrays) method was applied using TiGR (MeV, Version 4.8.1) [91].

### 4.14. qPCR

mRNA was reverse transcribed to cDNA using the iScript cDNA synthase kit (Bio-Rad) according to the manufacturer’s instructions. qPCR was performed with BioRad CFX Connect Real-Time PCR Detection System in triplicates using SsoAdvanced Universal SYBR Green Supermix. The normalized ΔΔCT values were calculated in the CFX Manager program. The following primers were used: Jag1 forward 5′ TGG TTG GCT GGG AAA TT 3′, Jag1 reverse 5′ TGG ACA CCA GGG CAC ATT C 3′, mHprt forward 5′ GCT TTC CTT GGT CAA GCA GTA CAG 3′, mHprt reverse 5′ GAA GTG CTC ATT ATA GTC AAG GGC ATA TCC 3′ [92].

### 4.15. Statistics

For analysis of paired kidneys, paired *t*-tests were applied using GraphPad Prism 7. For the quantification of unpaired kidneys, pHH3-positive cells and qPCR experiments, unpaired *t*-test was applied.

## Data Availability

Raw and processed RNA-seq data will be made available on GEO upon publication.

## Supplementary Materials

The following are available online at https://www.mdpi.com/article/10.3390/ijms22084157/s1, Figure S1: Ex vivo iron restriction as a model for metanephric development under iron-insufficient conditions, Figure S2: Ex vivo high glucose exposure does not influences long-term memory formation via DNA methylation at 30 mM glucose concentration, Figure S3: Anti-histamine drug Cyproheptadine leads to progenitor cell loss via inhibition of Wnt signaling. Table S1: RNA-seq analysis of high glucose kidney cultures.
