# Supplementary material for: Effects of Environmental Conditions on Nephron Number: Modeling Maternal Disease and Epigenetic Regulation in Renal Development
Source: Int J Mol Sci. 2021 Apr 16;22(8):4157. doi: 10.3390/ijms22084157 (PMC8073167; doi:10.3390/ijms22084157)
Supplement: Supplementary file 1 [file ijms-22-04157-s001.zip › Suppl files/Suppl Figures.pdf]

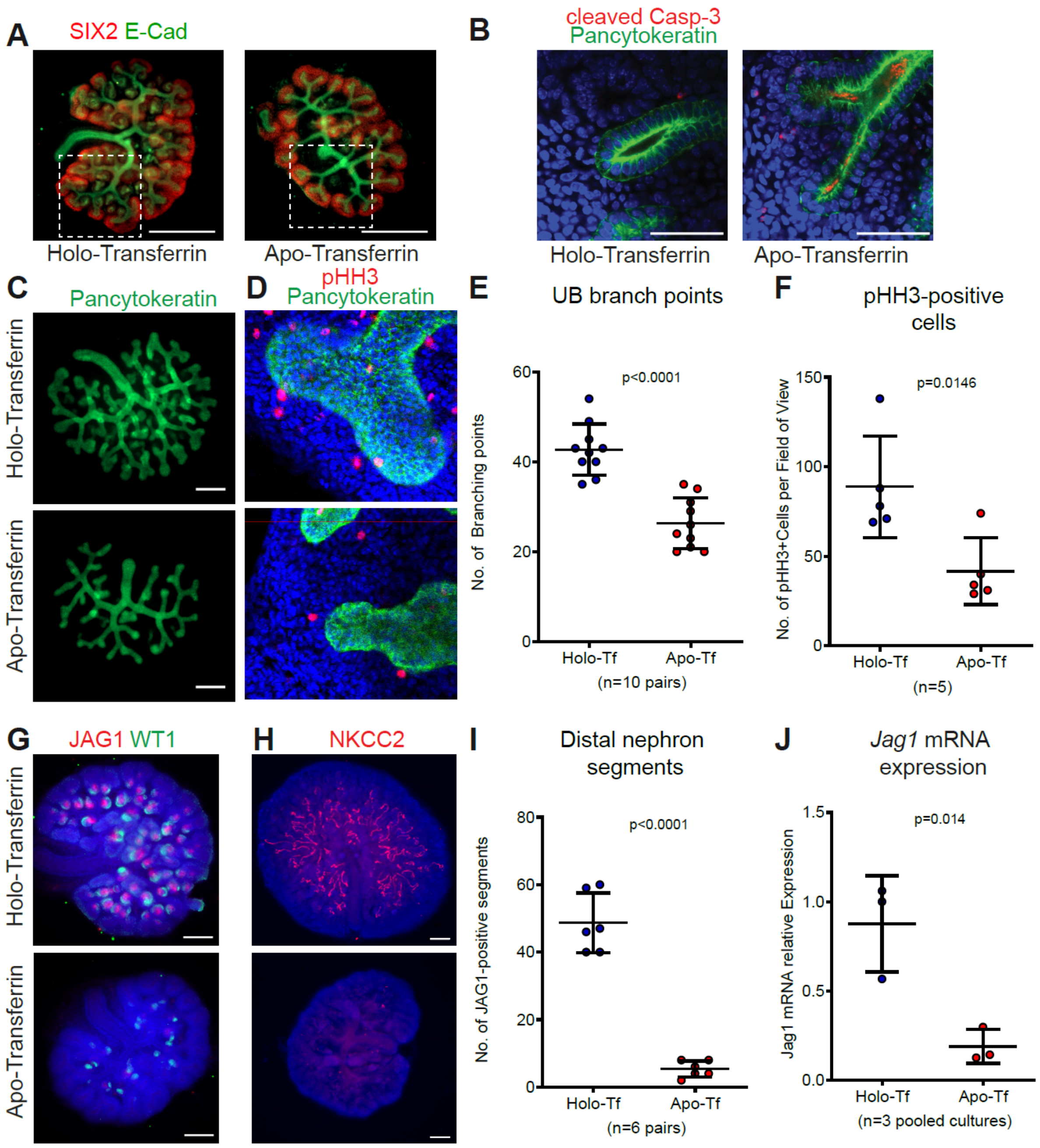

Supplementary Figure S1. ***Ex vivo*** **iron restriction as a model for metanephric development under iron-insufficient conditions.** A) Kidney cultures grown in holo-transferrin or apo-transferrin medium for 7 days stained for SIX2 and E-Cadherin. Images shown are representative in size and number of glomeruli. Scale bars: 500 µm. Dotted line: Magnification used for Figure 1H. B) Confocal images of holo-Tf and apo-Tf cultured explants stained against Pancytokeratin and cleaved caspase-3. Scale bar, 20 µm. C) Kidney cultures stained against Pancytokeratin and D) phospho-histone H3. E) Number of ureteric bud (UB) branch points and F) number of mitotic cells per field of view. n=5, unpaired t-test. error bars, mean ± SD. **, p-value = 0.0146. G) Widefield images of holo-Tf and apo-Tf cultured explant pairs stained against JAG1 and WT1 after 48h of culture to show early nephron morphology. Scale bars: 100 µm. H) Kidneys after 7 days in culture stained for distal tubules using NKCC2. Scale bar, 100 µm. I) JAG1-positive segments per explant after 48h of culture, n=6, paired t-test, mean ±SD.

J. *Jag1* mRNA relative to mHprt as evaluated by qPCR after 48h of culture, n=3 pooled cultures of 5 explants each, paired t-test, mean ±SD. **, p-value= 0.014.

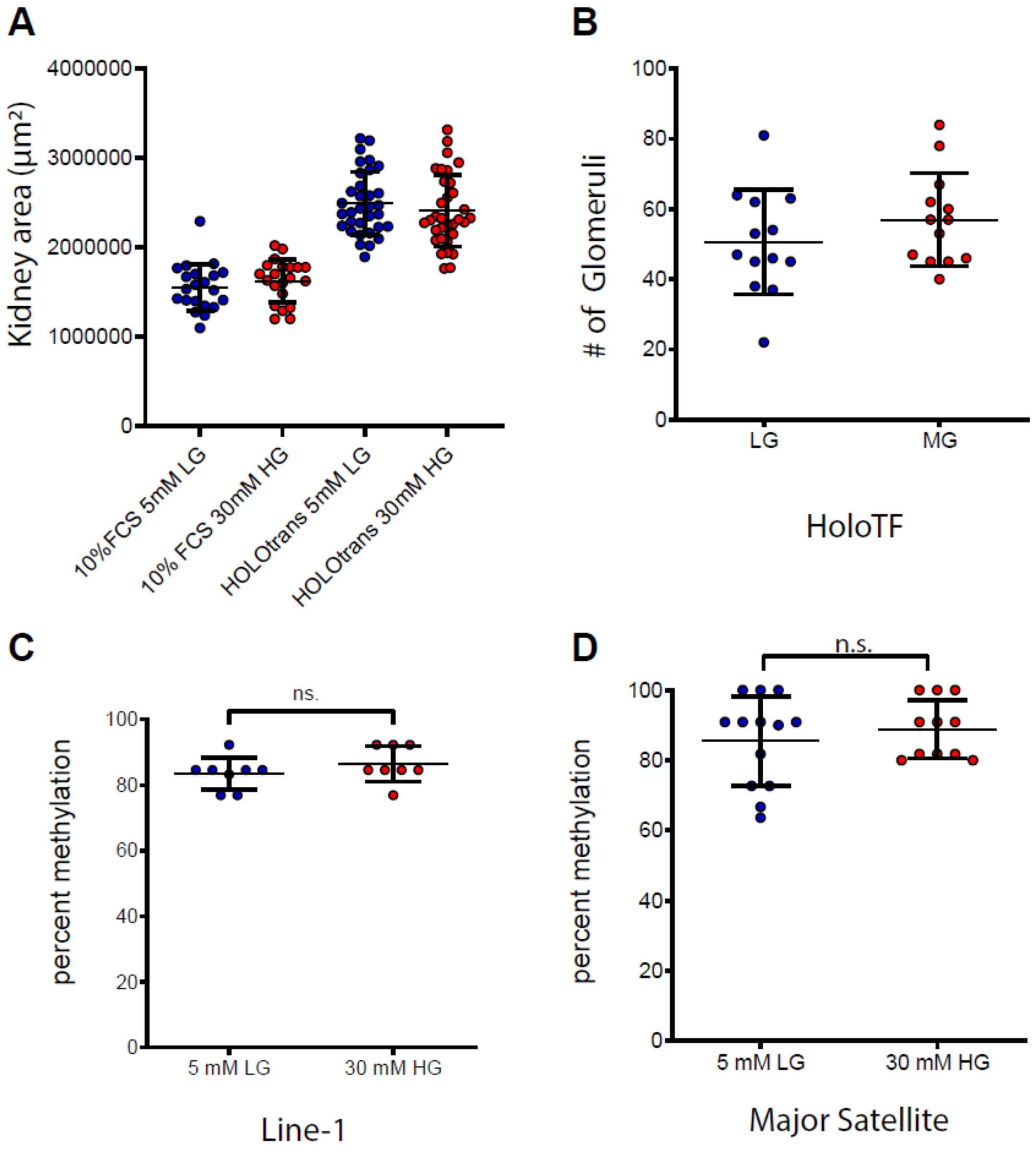

Supplementary Figure S 2. *Ex vivo* high glucose exposure does not influences long-term memory formation via DNA methylation at 30 mM glucose concentration. A) Measurement of kidney area after 7 days in low glucose medium (5.5 mM), or high glucose medium (30 mM) using different base media shows no difference in growth rate in the same base medium. B) Number of glomeruli is not decreased in HoloTf base medium between low and high glucose conditions. C) Analysis of DNA methylation at *LINE*-1 and D, *Major Satellite* loci shows no DNA hypomethylation in high glucose treated conditions. N.s., not significant.

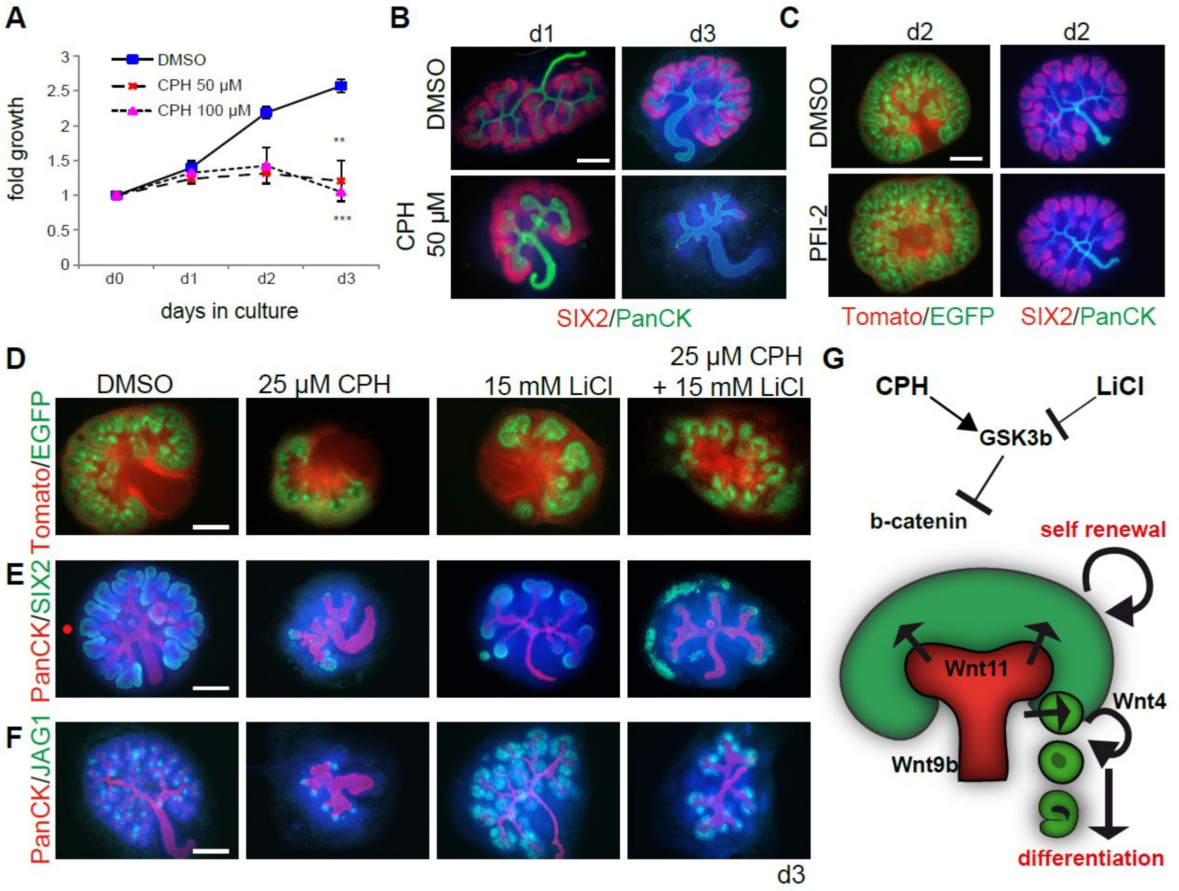

Supplementary Figure S3. Anti-histamine drug Cyproheptadine leads to progenitor cell loss via inhibition of Wnt signaling. A) Cyproheptadine (CPH) 50 and 100 µM concentrations lead to abrogated organ growth. B) Progenitor marker SIX2 is still present d1, but almost completely gone by d3 of culture with CPH inhibition. C) SET7/9 inhibitor PFI-2 did not lead to changes in renal organ development. D) Six2.Cre fluorescent reporter mice, E) SIX2 staining and F) JAG1 staining on control (DMSO), 25 µM CPH, LiCl or CPH and LiCl inhibition shows increased presence of SIX2-positive progenitor cells and JAG1-positive nephrons under of both CPH inhibition and GSK3b inhibitor LiCl, thus counteracting CPH activation of GSK3b. G) Schematic of WNT signalling and inhibitory mechanisms in nephron development.
